# Why We Die

**Published:** 1875-11

**Authors:** 


### WHY WE DIE.

Nearly one-fourth of all the deaths in Massachusetts last year were from consumption. The next greatest human destroyer was dysentery, commonly called *bloody flux.* Consumption is seated in the lungs,—dysentery is located about that portion of the bowels immediately under the stomach. Cough is the most universally observed symptom in consumption; passing blood is the inseparable attendant of dysentery. The spark which kindles up consumptive disease is sudden changes in the temperature of the body from a heat above what is natural to one that is below. The most universal causes of dysentery are breathing bad air, eating bad food, and drinking bad beverages.

The practical knowledge of these things and a possible and wise avoidance of them, would sweep from the list of human maladies the two deadliest of all diseases known to civilized life; and yet, not one in a dozen can be induced to wisely guard against cooling off too soon after exercise, or to avoid breathing an unwholesome and close atmosphere, and the swallowing of improper solids and fluids. The result of this ignorance and inattention, is, that the average of human life in the most intelligent State in the Union, and the thriftiest, does not exceed twenty-eight years, when it ought to exceed "*three score and ten!*"

---

THE best capital that a young man can start with in life is industry, good sense, courage, temperance, and an admiration for whatever is noble and good. They are better than cash, credit, or friends.

